# Draft genome sequence of the marine *Rhodobacteraceae* strain O3.65, cultivated from oil-polluted seawater of the Deepwater Horizon oil spill

**DOI:** 10.1186/s40793-016-0201-7

**Published:** 2016-10-13

**Authors:** Helge-Ansgar Giebel, Franziska Klotz, Sonja Voget, Anja Poehlein, Katrin Grosser, Andreas Teske, Thorsten Brinkhoff

**Affiliations:** 1 Institute for Chemistry and Biology of the Marine Environment (ICBM), University of Oldenburg, Oldenburg, Germany; 2 Department of Genomic and Applied Microbiology and Göttingen Genomics Laboratory, Institute of Microbiology and Genetics, University of Göttingen, Göttingen, Germany; 3 Department of Marine Sciences, University of North Carolina, Chapel Hill, NC USA

**Keywords:** *Rhodobacterales*, *Rhodobacteraceae*, *Roseobacter*, Oil spill, Deepwater Horizon, Oil degradation, Hydrocarbon, Marine bacteria

## Abstract

The marine alphaproteobacterium strain O3.65 was isolated from an enrichment culture of surface seawater contaminated with weathered oil (slicks) from the Deepwater Horizon (DWH) oil spill and belongs to the ubiquitous, diverse and ecological relevant *Roseobacter* group within the *Rhodobacteraceae*. Here, we present a preliminary set of physiological features of strain O3.65 and a description and annotation of its draft genome sequence. Based on our data we suggest potential ecological roles of the isolate in the degradation of crude oil within the network of the oil-enriched microbial community. The draft genome comprises 4,852,484 bp with 4,591 protein-coding genes and 63 RNA genes. Strain O3.65 utilizes pentoses, hexoses, disaccharides and amino acids as carbon and energy source and is able to grow on several hydroxylated and substituted aromatic compounds. Based on 16S rRNA gene comparison the closest described and validated strain is *Phaeobacter inhibens* DSM 17395, however, strain O3.65 is lacking several phenotypic and genomic characteristics specific for the genus *Phaeobacter*. Phylogenomic analyses based on the whole genome support extensive genetic exchange of strain O3.65 with members of the genus *Ruegeria*, potentially by using the secretion system type IV. Our physiological observations are consistent with the genomic and phylogenomic analyses and support that strain O3.65 is a novel species of a new genus within the *Rhodobacteraceae*.

**Electronic supplementary material:**

The online version of this article (doi:10.1186/s40793-016-0201-7) contains supplementary material, which is available to authorized users.

## Introduction

The *Roseobacter* clade is a major marine bacterial group, often associated with phytoplankton blooms [1–3], and accounts for up to 35 % of the bacterioplankton in coastal waters and the Southern Ocean [4–6]. The *Roseobacter* clade belongs to the family *Rhodobacteraceae* within the order *Rhodobacterales*, among the *Alphaproteobacteria* [7]; organisms of this group show a highly diversified range of physiological adaptations to various marine ecosystems [4, 5, 8]. Several taxa of this group are stimulated by different hydrocarbon compounds in laboratory experiments or in situ, suggesting a function in aerobic hydrocarbon degradation. Furthermore, pathways for oxygenic degradation of aromatic compounds and genes encoding for enzymes in alkane degradation were described for these bacteria [9]. Contributions of *Roseobacter*-related phylotypes to oil degradation were indicated by surveys using 16S rRNA gene based molecular biological techniques [10–14], but only a few studies were based on cultivation approaches [15, 16].

With this study, we fill this gap by specific isolation, genomic and physiological analysis of a bacterium of the *Roseobacter* clade isolated from seawater contaminated with weathered oil slicks from the Deepwater Horizon oil spill, one of the worst anthropogenic disasters in maritime petroleum production. Within 84 days (20^th^ April to 15^th^ July 2010) over 4.1 million barrels (~6.5x10^8^ L) of crude oil burst out into the Gulf of Mexico in a water depth of 1500 m [17]. Massive microbial community shifts were observed in the deep hydrocarbon plume at about 1,100 m depth, and in surface waters contaminated with slicks of weathered oil [12, 18–21].

Approximately two weeks after the beginning of the discharge, the first samples of oil slick-contaminated surficial seawater were collected, and were dominated by aromatic hydrocarbon degrading *Cycloclasticus* spp. and heterotrophic members of the *Alteromonadales* (*Pseudoalteromonas*, *Alteromonas* and *Colwellia* spp.) as well as members of the *Rhodobacteraceae* [20]. Passow and colleagues [22] reported that weathered crude oil slicks at the air-water interface were transformed into water-in-oil emulsions. These emulsions promoted the formation exopolymeric substances, mostly composed of polysaccharides; these coalesced into huge mucus-rich marine snow aggregates acting as hot spots for oil-specialized or -associated microbes, in which emulsified oil and EPS served as diverse food source for the highly active bacterial community [12, 23]. The bacterial communities associated with these aggregates included diverse phyla of *Gammaproteobacteria*, *Bacteroidetes*, and different organisms of the *Roseobacter* clade [12], and were distinctly different compared to those in the oil-contaminated water column [20].

The succession of different microbial taxa being abundant at distinct time points or steps during degradation of oil-derived hydrocarbons suggests a metabolic network comprising i) primary hydrocarbon-degrading and specialized microbes (involved in consumption, hydrolysis, oxidation of distinct hydrocarbons), ii) emulsifying microbes increasing the hydrocarbon bioavailability for the networkers, and iii) a very diverse group of secondary hydrocarbon consumers. All together form a complex assemblage of microbes involved in degradation of a wide spectrum of oil-derived hydrocarbons [12, 24].

Strain O3.65 was isolated from contaminated seawater of the DWH oil spill. Subsequent comparative analysis of the 16S rRNA gene sequences revealed that strain O3.65 belongs to the *Roseobacter* group, with *Phaeobacter* and *Ruegeria* species as closest described relatives. Here, we present a set of features and physiological characteristics of strain O3.65, and a description of the draft and annotated genome sequence of this organism. Furthermore, we partially elucidate its contribution in oil degradation and classify strain O3.65 into the above mentioned microbial oil degradation network based on the genomic and physiological analyses.

## Organism information

### Classification and features

Strain O3.65 was isolated from an enrichment culture of surface seawater sample contaminated with weathered oil from the DWH oil spill (Table 1). The sample was collected on June 1^st^ in 2010, and was subsequently stored undisturbed in a 50 ml Falcon tube for four years at 4 °C in the dark. The inoculum for isolation was taken from the underlying water–oil phase, directly below the oil layer (Additional file 1: Figure S1), and streaked out on agar plates (1.5 % w/v) containing 10 % marine broth (MB 2216, Difco) diluted with artificial seawater [25]. Plates were incubated at 20 °C in the dark until colonies were visible (2–5 days). For purification single colonies were picked and transferred at least three times to fresh plates with the same medium. Tests for purity of the culture, extraction of chromosomal DNA and sequencing of the 16S rRNA gene sequence were performed after Giebel et al. [26].

**Table 1:** Classification and general features of *Rhodobacteraceae* strain O3.65 according to the MIGS recommendations [92]

MIGS ID	Property	Term	Evidence code^a^
	Classification	Domain *Bacteria*	TAS [93]
		Phylum *Proteobacteria*	TAS [94]
		Class *Alphaproteobacteria*	TAS [95, 96]
		Order *Rhodobacterales*	TAS [95]
		Family *Rhodobacteraceae*	TAS [7, 95, 97]
		Genus *not specified*	
		Species *not specified*	
		(Type) strain: O3.65 (LPUY00000000.1)	
	Gram stain	negative	IDA
	Cell shape	rod shaped	IDA
	Motility	motile	IDA
	Sporulation	none	NAS
	Temperature range	mesophile	IDA
	Optimum temperature	30 °C	IDA
	pH range; Optimum	not specified	
	Carbon source	oligo-, di-saccharides, organic acids, amino acids, hydroxylated aromatic hydrocarbons	IDA
	Energy metabolism	heterotrophic	IDA
MIGS-6	Habitat	marine	IDA
MIGS 6.3	Salinity	1- < 8 %, optimum 3.5 %	IDA
MIGS-22	Oxygen requirement	aerobic	IDA
MIGS-15	Biotic relationship	unknown	NAS
MIGS-14	Pathogenicity	none	NAS
	Biosafety level	1	TAS [98]
MIGS-4	Geographic location	Gulf of Mexico	IDA
MIGS-5	Sample collection	June 1, 2010	IDA
MIGS-4.1	Latitude	28°43.967 N	IDA
MIGS-4.2	Longitude	88°22.993 W	IDA
MIGS-4.4	Altitude	not specified	

^a^Evidence codes - *IDA* inferred from direct assay, *TAS* traceable author statement (i.e., a direct report exists in the literature), *NAS* non-traceable author statement (i.e., not directly observed for the living, isolated sample, but based on a generally accepted property for the species, or anecdotal evidence). These evidence codes are from the Gene Ontology project [99]

**Fig. 1:**
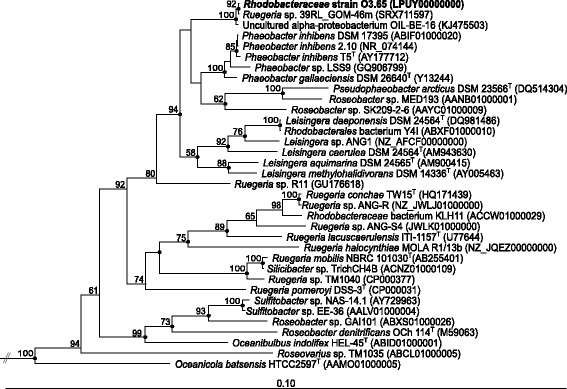
Phylogenetic tree highlighting the position of *Rhodobacteraceae* strain O3.65 relative to other genome sequenced and type strains within the genera *Phaeobacter*, *Pseudophaeobacter*, *Ruegeria*, *Leisingera* and additional strains of the *Rhodobacteraceae*. The tree was inferred from nearly full-length 16S rRNA gene sequences (≥1300 bp) using the neighbour joining tool of the ARB software [28]. Only bootstrap values ≥50 % (derived from 1000 replicates) are shown. Filled circles indicate nodes also recovered reproducibly with maximum-likelihood (RAxML) calculation. Strains and their corresponding GenBank accession numbers are listed in Additional file 1: Table S1. All strains in the tree are genome sequenced, except clone Oil-BE-016 (KJ475503). Type strains are designated by ^T^. Three *Synechococcus* strains (AY946243, CP000951, AF448073) served as outgroup (not shown)

Comparison of the 16S rRNA gene sequence of strain O3.65 with those of type strains of the *Rhodobacteraceae* was performed using the Blast search tool of the National Center for Biotechnology Information [27]. For phylogenetic analysis and similarity matrix calculation we used the ARB software [28]. The tree in Fig. 1 comprises all currently available genome sequenced *Phaeobacter*, *Pseudophaeobacter*, *Leisingera* and *Ruegeria* strains, covering most of the type strains and species of those groups and additional genome-sequenced species of the *Roseobacter* group.

**Fig. 2:**
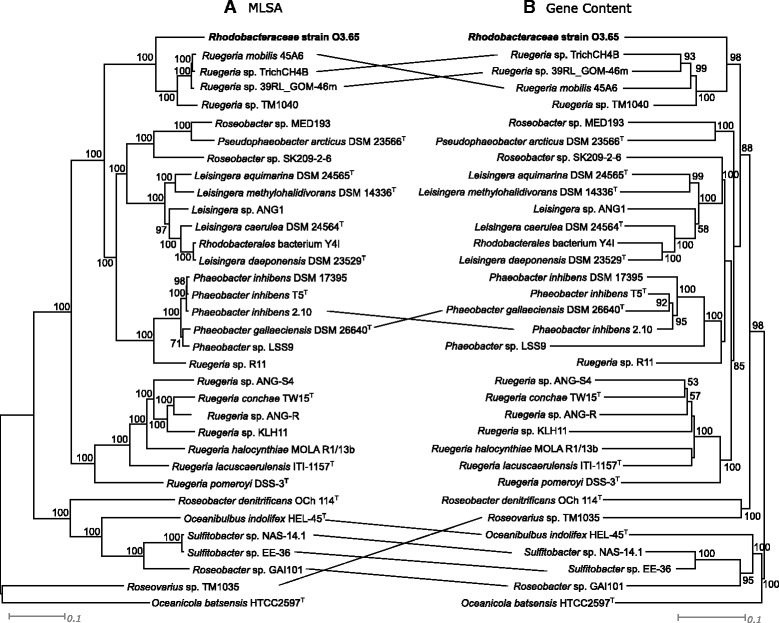
Tanglegram of genome based trees. **a** Maximum likelihood tree based on genomic data of organisms affiliated with the genera *Phaeobacter*, *Pseudophaeobacter*, *Ruegeria*, *Leisingera* and additional strains of the *Roseobacter* clade inferred with 500 bootstraps (BS) with RAxML after Stamatakis (2014) [100]. The alignment was created from 684 orthologous single-copy genes present in all genomes (Multilocus Sequence Analysis; MLSA) after total protein sequences of the genomes were extracted from the corresponding GenBank files and used for the downstream analysis with an in house pipeline at the Goettingen Genomics Laboratory (J. Vollmers, unpubl.). In brief, clusters of orthologs were generated using proteinortho version 5 [101], inparalogs were removed, the remaining sequences were aligned with MUSCLE [102] and poorly aligned positions automatically filtered from the alignments using Gblocks [103]. **b** Gene content tree including singletons of the same organisms as in A based on an orthologs-content matrix representing presence or absence of a gene in a certain genome, inferred with Neighbour Joining (1000 BS). Both scripts for this pipeline, PO_2_MLSA.py and PO_2_GENECONTENT.py, are available at github. Numbers at the nodes specify BS values ≥50 %. Scale bars represent 10 % sequence divergence. For Genbank accession numbers see Additional file 1: Table S1. For a clear view only lines were given linking the same species at different positions

Based on the 16S rRNA gene sequences a greater monophyletic cluster, supported by a high bootstrap value of 94 %, was obtained encompassing strain O3.65 and related sequences, as well as the genera *Phaeobacter*, *Pseudophaeobacter* and *Leisingera*. Strain O3.65 forms a subcluster together with the undescribed strain *Ruegeria* sp. 39RL_GOM-46 m (SRX711597) isolated from an oil-amended biotrap, and the clone Oil-BE-016 (KJ475503, [12]) obtained from an oil slick sample after lab incubation, both from the DWH oil spill and having a sequence similarity of 100 and 99 %, respectively. The 16S rRNA gene sequence of strain O3.65 shows minimal dissimilarities to those of its closest described and validated relatives, i.e. 1.6 % to *Phaeobacter inhibens* DSM 17395 and 1.7 % to both type strains *P. gallaeciensis* DSM 26640^T^ and *Phaeobacter inhibens* T5^T^. Dissimilarity values increased up to 1.9 or higher for type species of the genera *Ruegeria*, *Leisingera* and *Pseudophaeobacter* (Additional file 1: Table S1; [12, 29–58]). Despite these low dissimilarity values, classification of strain O3.65 as a new *Phaeobacter* species was not supported by phylogenetic analysis only on 16S rRNA gene level (Fig. 1). The clearly separated subcluster of strain O3.65 leads to the assumption that this organism represents a new phylogenetic lineage at the species and genus level. Comparative analysis of genomic data (see below, Fig. 2) supports a classification as a new genus within the *Rhodobacteraceae*. The multitude of recent reclassifications of species within the *Phaeobacter*-*Leisingera* group [29–31, 59] shows the difficulty of accurate classification of (new) species related to these closely related genera. Furthermore, we suggest reclassification of strain *Ruegeria* sp. 39RL_GOM-46 m based on a coherent description and validation of strain O3.65 as member of a new genus in the future.

Besides strain O3.65, we isolated similar organisms with the same 16S rRNA gene sequence from agar plates inoculated with oil-polluted seawater from another sample taken at a different station after the DWH oil spill (data not shown). Furthermore, two independent studies found previously the same phylotype of strain O3.65 (SRX711597) and a second phylotype very similar (1382/1383 identities, [12]) to strain O3.65 in the Gulf of Mexico (see above). Therefore we conclude that strain O3.65 represents a physiologically and ecologically relevant ecotype for the DWH oil spill.

**Fig. 3:**
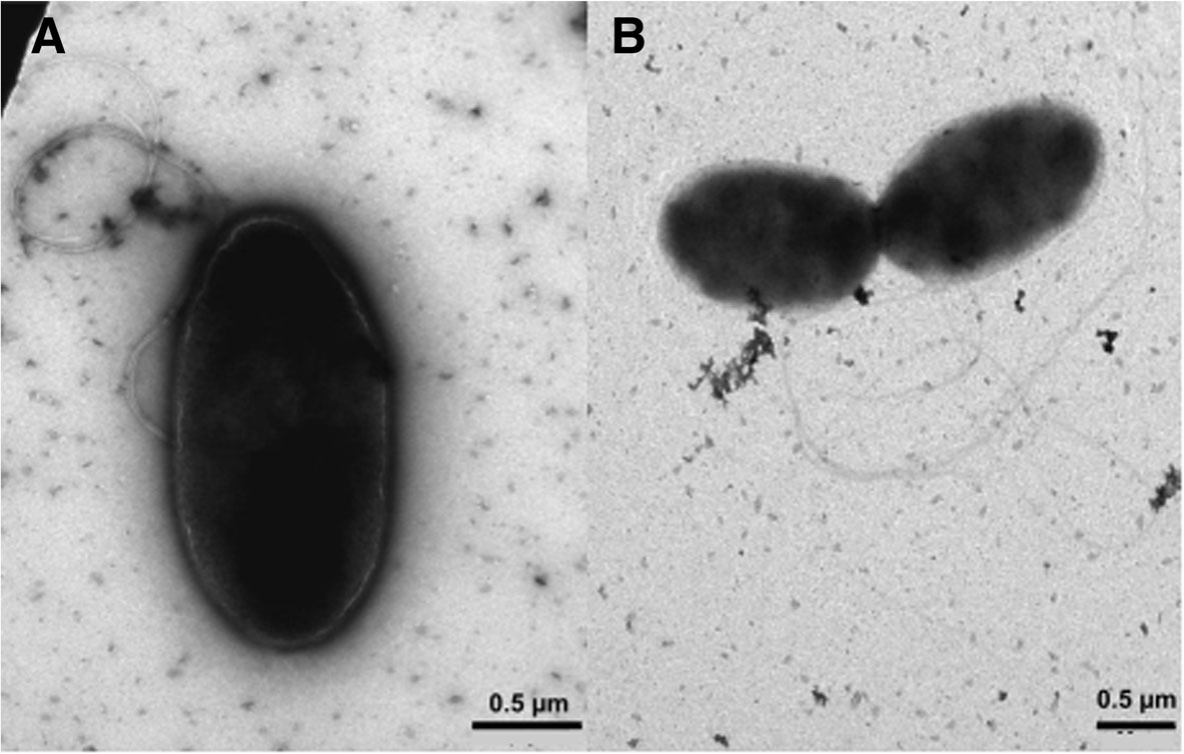
Transmission electron micrographs of *Rhodobacteraceae* strain O3.65. **a** The typical rod-shaped morphology of a single cell with intact bundle of flagella, and (**b**) two cells by binary fission and their flagella. Cells were negatively stained. Scale bars 0.5 μm

Cells of strain O3.65 are ovoid rods, with a length of 1.3–2.2 μm and a width of 0.6–1.0 μm (Fig. 3). Cells are motile by means of a polar flagellum. O3.65 is a Gram-negative, marine, aerobic, mesophilic bacterium with an optimal growth temperature between 30 and 35 °C and an optimal salinity between 2.5 and 5 %. On Difco Marine Broth (MB) 2216 agar (Becton Dickinson, MD, USA) strain O3.65 forms smooth, shiny and convex colonies with regular edges of white to light beige color. Strain O3.65 utilizes pentoses, hexoses and disaccharides [(+)-L-arabinose, (+)-D-xylose, (−)-D-ribose, (+)-D-glucose (−)-L-fucose, (−)-D-fructose, (+)-D-cellobiose, (+)-D-sucrose;1 g/l final] as well as most amino acids (L-forms of alanine, aspartic acid, glutamic acid, histidine, arginine, threonine, tryptophane, phenylalanine, proline, leucine, valine; 1 mM final) as carbon and energy sources. Strain O3.65 is able to grow on several aromatic compounds, i.e., 4-hydroxy-benzoic acid, 3,4-dihydroxy-benzoic acid, p-coumarin, ferulic acid, tryptophan and vanillin.

## Genome sequencing information

### Genome project history

The genome of strain O3.65 was selected for sequencing based on its phylogenetic affiliation with the ecologically important and worldwide distributed *Roseobacter* clade and the lack of roseobacteral genomes in the course of studies on oil degradation of the DWH oil spill. The genome sequence was completed on February 18^th^, 2015, and presented for public access on January 19^th^, 2016. The genome project was deposited in the Genomes OnLine Database (GOLD) as project Gp0111538. The Whole Genome Shotgun project has been deposited at DDBJ/EMBL/GenBank under the accession number LPUY00000000.1. The version described in this paper is version 1. Table 2 presents a summary of the project information.

**Table 2:** Project information for *Rhodobacteraceae* strain O3.65

MIGS ID	Property	Term
MIGS-31	Finishing quality	Draft
MIGS-28	Libraries used	Nextera xt
MIGS-29	Sequencing platforms	Illumina GAiix
MIGS-31.2	Fold coverage	71.5x
MIGS-30	Assemblers	SPAdes v3.5
MIGS-32	Gene calling method	Prodigal v2.50
	Genome Database release	IMG; 2608642179
	Genbank ID	LPUY00000000.1
	Genbank Date of Release	January 19th, 2016
	GOLD ID	Gp0111538
	BIOPROJECT	PRJNA305382
MIGS-13	Source Material Identifier	O3.65
	Project relevance	environmental

### Growth conditions and DNA preparation

Strain O3.65 was grown at 20 °C in marine broth (MB2216, Difco) in the dark to the late exponential phase. Cells were harvested by centrifugation (10 000 g at 4 °C for 20 min) and subsequent DNA extraction was performed using a Power Soil DNA Isolation Kit (MoBio) according to the manufacturer’s specifications. The protocol includes bead beating for mechanical as well as chemical methods for cell lysis. A total of 1.3 μg of DNA was obtained.

### Genome sequencing and assembly

Whole-genome sequencing was performed using Illumina technology. Preparation of a paired-end sequencing library with the Illumina Nextera XT library preparation kit and sequencing of the library using the Genome Analyzer IIx were performed as described by the manufacturer (Illumina, San Diego, CA, USA). A total of 4.6 million paired-end reads were derived from sequencing and trimmed using Trimmomatic version 0.32 [60]. De novo assembly of all trimmed reads with SPAdes version 3.5.0 [61] resulted in 125 contigs and 71.5-fold coverage.

### Genome annotation

Protein-coding genes were identified as part of the genome annotation pipeline of the Integrated Microbial Genomes platform using Prodigal v2.50. The predicted CDS were translated and used to search the CDD, KEGG, UniProt, TIGRFam, Pfam and InterPro databases. These data sources were combined to assert a product description for each predicted protein. Non-coding genes and miscellaneous features were predicted using tRNAscan-SE [62], RNAmmer [63], Rfam [64], TMHMM [65] and SignalP [66]. Additional gene prediction analyses and functional annotation were performed within the IMG-ER platform [67].

## Genome properties

The genome statistics are provided in Table 3. The draft genome of strain O3.65 consists of 125 scaffolds with a total length of 4,852,484 bp and an overall G + C content of 61.50 %. Of the 4,654 predicted genes, 4591 (98.65 %) are protein-coding, and 63 are RNA genes. No pseudogenes or CRISPR counts were found. Most of the protein-coding genes (71 %) were assigned to putative functions. Besides the chromosome we assume strain O3.65 is carrying at least five extrachromosomal elements derived from five different typical plasmid repABC-type replication modules, commonly found within the *Rhodobacteraceae* [68]. The number and length of scaffolds of this draft genome did not allow a detailed view on plasmid organization. The distribution of genes into COGs functional categories is listed in Table 4.

**Table 3:** Nucleotide content and gene count levels of the draft genome of *Rhodobacteraceae* strain O3.65

Attribute	Genome (total)	
	Value	% of total
Genome size (bp)	4,852,484	100.00
DNA coding (bp)	4,330,569	89.25
DNA G + C (bp)	2,984,418	61.50
DNA scaffolds	125	
Total genes	4,654	100.00
Protein-coding genes	4,591	98.65
RNA genes	63	1.35
Pseudo genes	0	
Genes in internal clusters		
Genes with function prediction	3,868	83.11
Genes assigned to COGs	3,308	71.08
Genes assigned to pfam domains	3,953	84.94
Genes with signal peptides	390	8.38
Genes with transmembrane helices	991	21.29
CRISPR repeats	0

**Table 4:** Number of genes associated with the 25 general COG functional categories of *Rhodobacteraceae* strain O3.65

Code	Value	%age	Description
J	193	5.19	Translation, ribosomal structure and biogenesis
A	n.a.	n.a.	RNA processing and modification
K	300	8.07	Transcription
L	110	2.96	Replication, recombination and repair
B	3	0.08	Chromatin structure and dynamics
D	39	1.05	Cell cycle control, Cell division, chromosome partitioning
V	n.a.	n.a.	Defense mechanisms
T	58	1.56	Signal transduction mechanisms
M	135	3.63	Cell wall/membrane biogenesis
N	183	4.92	Cell motility
U	64	1.72	Intracellular trafficking and secretion
O	1	0.03	Posttranslational modification, protein turnover, chaperones
C	11	0.3	Energy production and conversion
G	83	2.23	Carbohydrate transport and metabolism
E	156	4.19	Amino acid transport and metabolism
F	249	6.7	Nucleotide transport and metabolism
H	346	9.3	Coenzyme transport and metabolism
I	393	10.57	Lipid transport and metabolism
P	91	2.45	Inorganic ion transport and metabolism
Q	183	4.92	Secondary metabolites biosynthesis, transport and catabolism
R	216	5.81	General function prediction only
S	218	5.86	Function unknown
-	135	3.63	Not in COGs

*Abbreviation: n.a*. not assigned

The total is based on the total number of protein coding genes in the genome

## Insights from the genome sequence

Several pathways in the aerobic hydrocarbon degradation by ring modifications and alkane hydroxylases are known and used by members of the *Roseobacter* group [9]. Yet, analysis of genomic homology could be difficult due to the low amount of gene synteny among genomes of strains even on species level, and the high distribution of functionally related genes across multiple loci [69]. In general, strain O3.65 is not able to hydroxylate an aromatic ring via specific ring hydroxylating dioxygenases, such as benzoate 1,2-dioxygenase or naphthalene 1,2-dioxygenase; genes of the protein families 00355, 00848 and 00866 were not found [9]. The draft genome of strain O3.65 is carrying none or only a low number of genes (given in parentheses) encoding for enzymes involved in the cleavage of gentisate (gdo; 0), the benzoyl-CoA pathway (box; 0) and the meta cleavage of homoprotocatechuate (hgd; 2 of 7). In contrast, strain O3.65 does contain several putative ring-cleaving dioxygenases: Two aromatic ring-opening dioxygenases, catalytic subunit, LigB family (TRIHO_09370; TRIHO_18120; pfam02900), hydroquinol and 1,2-catechol dioxygenases (TRIHO_05060; TRIHO_09430; pfam04444/pfam 00775), protocatechuate 3,4-dioxygenase alpha and beta subunit (TRIHO_21670/60; pfam00755) and at least four catechol 2,3-dioxygenases (TRIHO_03150; TRIHO_07560; TRIHO_29300; TRIHO_43160 pfam00903, TRIHO_09100; TRIHO_20770 pfam12681). All those ring-cleaving enzymes are essential for degrading substances like protocatechuate, vanillin, 4-hydroxybenzoate, ferulic acid or p-coumarin, which is consistent with our growth experiments (see discussion of morphology and physiology above).

However, genes for degradation of hydroxylated aromatic compounds like p-hydroxybenzoate via protocatechuate (pca, β-ketoadipate pathway) are present in the genome of O3.65. For example, the genes *pobA* and *pcaDCHGB* (TRIHO_21630-80) are homologues to genes found in *Silicibacter* sp. TM1040 and *Ruegeria mobilis* 45A6. The genes *pcaIJ* (TRIHO_43620/30) of strain O3.65 coding for the 3-oxoadipate:succinyl CoA transferase are arranged in the same way as in *Citreicella* sp. SE45, but the entire neighboring gene arrangement of both strains differs completely from those of other *Roseobacter* representatives. Comparative analysis shows that all *Phaeobacter*, *Pseudophaeobacter*, *Leisingera* and *Ruegeria* spp. do not have the genes *pcaIJ* for an 3-oxoadipate:succinyl CoA transferase (EC 2.8.3.6); instead, it seems to be replaced by an 3-oxoacid CoA-transferase (EC 2.8.3.5) with an AA-composition similarity of 32 %. Also missing for the above mentioned genomes, but present for strain O3.65 and located next to the subunit *pcaIJ*, is a regulatory protein (coded by *pcaR*; 2609025149, TRIHO_43610) needed for functionality of the enzyme 3-oxiadipate CoA transferase. PcaR, characterized for *Pseudomonas putida* [70] was blasted against the *Phaeobacter-Leisingera*-group finding genes with ~30 % similarity, but in distinctly different neighborhoods than in O3.65, which could imply other functions of the IclR family (transcriptional regulator, Pfam01614) to which pcaR belongs. Moreover, no similar *pcaR*-genes were found in any genomes of *Ruegeria* spp., underlining its distinctiveness from these two groups. We assume that strain O3.65 is able to metabolize phenylacetic acids via the phenylacetyl-CoA pathway (paa) having all the necessary genes (*paaABCDE*), except the catalytic subunit. However, strain O3.65 is able to grow on phenylalanine, which is degraded via the paa-pathway, like in *P. inhibens* DSM 17395 [71]. Besides, strain O3.65 is able to carry out the degradation of the aromatic intermediate homogentisate by a specific homogentisate 1,2-dioxygenase (TRIHO_32660; pfam04209).

Even though strain O3.65 is carrying the gene for an alkane 1-monooxygenase (pAH1; coded by *alkB* locus tag TRIHO_03510) and all genes for the following pathway steps for metabolizing an alkane into a fatty acid, it did not exhibit any growth in experiments on nonane, decane, hexadecane or paraffin. In contrast, *Pseudophaeobacter arcticus* DSM 23566^T^ was able to grow on all those alkanes. Maybe this is caused by the missing gene coding for rubredoxin reductase (EC 1.18.1.1/4) in strain O3.65, required for the reducing step of rubredoxin. Rubredoxin and rubredoxin reductase are essential electron transfer proteins and present in known alkane degraders like *Alcanivorax dieselolei* B5 [72]. Notably, this gene is also missing in strain DSM 23566^T^, leading to the conclusion that there might be other ways of alkane degradation, as already stated by Buchan and Gonzalez (2010) [9]. Perhaps EPS [73] or unknown substances from other oil degrading bacteria in contaminated seawater could help solubilizing oil substances, what has to be shown for strain O3.65. If this can be confirmed, strain O3.65 is involved in the microbial degradation of n-alkanes, which were found in enhanced concentrations in the oil-slick as well as polycyclic aromatic hydrocarbons of high-molecular weight [18, 74, 75].

In summary, we observed that strain O3.65 is able to degrade several oil-derived compounds via different pathways for hydrocarbon degradation. However, the missing pathways, especially the missing RHD, indicate that strain O3.65 does not belong to the group of specialized primary oil-degrading microbes within the hydrocarbon-degrading metabolic network. Instead, strain O3.65 belongs to the group of secondary hydrocarbon consumers feeding on special oil-derived components, i.e. “predigested” hydrocarbon fragments or on non-oil exudates from primary oil degraders. Matching to this was the found of an *Alcanivorax* affiliated isolate in our sample (unpublished data), which are well-known primary petroleum degraders, commonly rising in numbers during oil spills [76].

Using a whole genome comparison approach by multilocus sequence analysis, based on 684 orthologous single-copy genes and by gene content analysis of the same strains considered as in the 16S rRNA gene analysis above, separate clustering of strain O3.65 is supported (Fig. 2). By MLSA and gene content analysis, the closest related genus of strain O3.65 is not *Phaeobacter* (Fig. 1) but *Ruegeria*, supported by bootstrap values of 100 and 98 %, respectively. Four *Ruegeria* strains (*R.* sp. 39RL_GOM-46 m, *R. mobilis* 45A6, *R.* sp. TrichCH4B and *R.* sp. TM1040), separated from other *Ruegeria* spp., form the sequence cluster adjacent to strain O3.65. While *Ruegeria* sp. 39RL_GOM-46 m was obtained from the same oil-polluted environment and has an identical 16S rRNA gene sequence (Fig. 1), the MLSA or the gene content approach separate this strain from strain O3.65, and indicate a different genetic potential and evolution of both strains. The other three closely related *Ruegeria* strains have a 16S rRNA gene dissimilarity of 3.5 % and 4.6 %, respectively. Strains affiliated to *Phaeobacter*, *Pseudophaeobacter* and *Leisingera* clustered separately within the single genera in distinct groups, at which their clustering pattern is nearly identical by both calculation methods, emphasizing a high stability of the phylogenetic analyses.

Furthermore, we compared all available genome-sequenced *Phaeobacter*, *Pseudophaeobacter*, *Leisingera* and *Ruegeria* strains covering most of the type strains as well as type species of those genera and the draft genome of strain O3.65 by *in silico* DNA-DNA hybridization using the online tool genome to genome distance calculator (GGDC 2.0; [77–79]). The DDH similarities of strain O3.65 to the above mentioned reference strains are listed in Additional file 1: Table S1. The highest similarity was found for the genome of strain *Ruegeria* sp. 39RL_GOM-46 m with a maximal value of 100 ± 0.1 % implying that strain 39RL_GOM-46 m is another strain of a new proposed species represented by *Rhodobacteraeae* strain O3.65. This high similarity is in agreement with the 16S rRNA gene sequence similarity. Further, the GGDC analysis revealed a distinctly low mean similarity of the O3.65 genome (20.5 ± 4.8 %) compared to all other genomes considered in our study, including the other three closely related *Ruegeria* strains clustering together with strain 39RL_GOM-46 m and all available genome sequenced types species/strains of the genera *Phaeobacter*, *Pseudophaeobacter*, *Leisingera* and *Ruegeria*. This low similarity on genome level indicates a significant different genomic repertoire of strain O3.65 compared to its most closely-related neighbors, supporting that strain O3.65 represents a new species of a new genus within the *Rhodobacteraceae*, not distinguishable by 16S rRNA gene phylogeny only.

While aerobic anoxygenic photosynthesis is a widespread but phylogenetically dispersed feature among the *Roseobacter* group [8] strain O3.65 is not able to use light via aerobic anoxygenic photosynthesis or rhodopsins. However, both types of the *coxL* gene for the carbon monoxide dehydrogenase are present, implying a role within the marine carbon monoxide cycling, because only strains with both *coxL* forms (I and II; TRIHO_01790-60 and TRIHO_28700-40) are able to oxidize carbon monoxide [80, 81]. This could provide an additional energy source for strain O3.65 not available for other non-chemolithotrophic microbes [82].

Some *Roseobacter* species are able to synthesize the essential cofactor biotin, e.g. *P. gallaeciensis* BS107 and *Ruegeria* sp. R11 [83]. No genes for biotin synthesis were found in the genome of strain O3.65, as shown previously for *Ruegeria* sp. TM1040 and *R. pomeroyi* DSS-3 [83]. Therefore, bacteria missing the synthesis apparatus of biotin are equipped with a highly affine (or high-affinity) biotin uptake system present in strain O3.65, and homologous to those in *Leisingera caerulea* DSM 24564^T^ and *Leisingera methylohalidivorans* MB2^T^/DSM 14336^T^.

An *in silico* analysis for secondary metabolites via the online tool antiSMASH 3.0 [84] revealed secondary metabolite clusters for bacteriocin, lassopeptide, ectoine and a type 1 polyketide synthase (PKS). PKSs mediate the biosynthesis of bioactive natural substances and are known for the genus *Phaeobacter* [85]. Genes encoding for iron-chelating siderophore biosynthesis and transport, commonly found in *Phaeobacter* and *Leisingera* species [29, 32, 33], are also present in genome of strain O3.65. The operon for biosynthesis (TRIHO_27280) is homologous to those in *P. inhibens* T5^T^ and the *Ruegeria* sp. strains TrichCH4B and TM1040. The operon coding for the uptake of siderophores (TRIHO_36570) is homolog to those in *R. mobilis* 45A6 and *Ruegeria* sp. TrichCH4B. Strain O3.65 is lacking genes coding for AHL synthetase proteins, described for *P. inhibens* T5^T^ [29] and *P. gallaeciensis* DSM 26640^T^ [34]. Moreover, the AHL synthetase protein was found in all genomes of the type strains of the *Leisingera*, *Pseudophaeobacter* and *Ruegeria* group listed in this study (Additional file 1: Table S1) with the exception of *R. mobilis* NBRC101030^T^. Several *Phaeobacter* strains [35, 85–89], including the *P. inhibens* strains DSM 17395 and T5^T^ as next described species to strain O3.65, are able to produce the antibiotic TDA and a brownish pigment [85]. These *Phaeobacter*-typical characteristics were not found to be encoded in the genome of strain O3.65 and could not be observed phenotypically.

Strain O3.65 is carrying at least three operons for the secretion system type IV (*virB*), which are versatile and involved in conjugation, DNA uptake or in effector translocation [90]. (TRIHO_37480, homolog to *Roseovarius* sp. 217, TRIHO_40140, TRIHO_41580 homolog to *Oceanibulbus indolifex* HEL-45^T^). Furthermore, genes for the flp pilus type IV are present in genome of strain O3.65, known to play important roles in surface adhesion, biofilm formation, motility, conjugation, and DNA transfer and uptake, with significant effects for pathogenicity [91] (TRIHO_20800 homolog to *R. mobilis* 45A6 and *Ruegeria* sp. TrichCH4BTRIHO_30860 homolog to *R. mobilis* 45A6 and *Ruegeria* sp. TM1040).

Our data of the draft genome revealed a diverse composition of several genes and functional operons of strain O3.65 originated from different phylogenetic groups, which was derived by their homologies. Having both opportunities to exchange or uptake DNA by pilus and secretion systems could be an explanation for carrying such a brought mixture of *Ruegeria*-, *Phaeobacter*- and *Leisingera*-like genes. Besides, this could elucidate the discrepancy of the phylogenetic classification based on 16S rRNA gene sequences and the genome based approaches (Figs. 1 and 2, see above).

## Conclusion

The differences detected based on the genomic and physiological data of strain O3.65 compared to previously described organisms within the *Rhodobacteraceae* *,* especially to the genus *Phaeobacter*, suggests that strain O3.65 represents a member of a new species within a new genus. The multitude of recent reclassifications of several strains within the *Rhodobacteraceae*, especially within the genera *Phaeobacter* and *Leisingera* [29–31, 59] shows the difficulty to accurately classify (new) species related to these phylogenetic clades based only on 16S rRNA gene level, and supports our suggestion of a new genus to avoid a misleading phylogenetic classification a priori. Strain O3.65 is lacking several features typical for the genus *Phaeobacter*, e.g. production of the antibiotic TDA and AHLs, pigmentation, the hgd-pathway and biotin synthesis. Even though based on 16S rRNA gene comparison the closest described strain is *Phaeobacter inhibens* DSM 17395, high genetic exchange of strain O3.65 with members of the genus *Ruegeria* is indicated by the MLSA and gene content analysis based on whole genome information. Strain O3.65 is able to degrade hydroxylated aromatic compounds by several pathways, but is lacking genes to utilize alkanes. However, strain O3.65 represents a new, abundant and ecologically relevant microbial species within the hydrocarbon degrading microbial community of the DWH oil spill. We assume that strain O3.65 belongs to the group of secondary hydrocarbon consumers feeding on special oil-components, on “predigested” hydrocarbon fragments, or on non-oil exudates from primary oil degraders.

## Additional file

Additional file 1: Figure S1. Enrichment culture of surface seawater contaminated with weathered oil (slicks) from the Deepwater Horizon (DWH) oil spill with oil and oil–water phase. From the latter, indicated by an arrow, Rhodobacteraceae strain O3.65 was isolated. **Table S1.** Dissimilarity (%) based on 16S rRNA gene sequence comparison 29 and in silico DNA-DNA hybridization (DDH) of strain O3.65 using 16S rRNA gene sequences and genomes of (typeT31) strains of the genera Phaeobacter, Pseudophaeobacter, Leisingera, Ruegeria and other relevant strains. The neighbor-joining distance matrix tool of the ARB software was used for calculation of 16S rRNA gene similarity. DDH was done using the genome to genome distance calculator (GGDC 2.0, DSMZ, http://ggdc.dsmz.de/distcalc2.php, [77, 78]) and represents values of the recommended formula 2 [79]. Table sorted by increasing dissimilarity. (PDF 262 kb)

## Abbreviations

AHL: N-acyl-L-homoserine lactone
DDH: DNA-DNA hybridization
DWH: Deepwater Horizon
EPS: Exopolymeric substances
MLSA: Multilocus sequence analysis
RHD: Ring hydroxylating dioxygenase
TDA: Tropodithietic acid
