# Supplementary material for: Draft genome sequence of the marine Rhodobacteraceae strain O3.65, cultivated from oil-polluted seawater of the Deepwater Horizon oil spill
Source: Stand Genomic Sci. 2016 Oct 13;11:81. doi: 10.1186/s40793-016-0201-7 (PMC5064897; doi:10.1186/s40793-016-0201-7)
Supplement: Additional file 1: Figure S1. — Enrichment culture of surface seawater contaminated with weathered oil (slicks) from the Deepwater Horizon (DWH) oil spill with oil and oil–water phase. From the latter, indicated by an arrow, Rhodobacteraceae strain O3.65 was isolated. Table S1. Dissimilarity (%) based on 16S rRNA gene sequence comparison 29 and in silico DNA-DNA hybridization (DDH) of strain O3.65 using 16S rRNA gene sequences and genomes of (typeT31) strains of the genera Phaeobacter, Pseudophaeobacter, Leisingera, Ruegeria and other relevant strains. The neighbor-joining distance matrix tool of the ARB software was used for calculation of 16S rRNA gene similarity. DDH was done using the genome to genome distance calculator (GGDC 2.0, DSMZ, http://ggdc.dsmz.de/distcalc2.php, [77, 78]) and represents values of the recommended formula 2 [79]. Table sorted by increasing dissimilarity. (PDF 262 kb) [file 40793_2016_201_MOESM1_ESM.pdf]

Supplemental data for

Draft genome sequence of the marine *Rhodobacteraceae* strain O3.65, cultivated from oil-polluted seawater of the Deepwater Horizon oil spill

Helge-Ansgar Giebel[1*], Franziska Klotz[1], Sonja Voget[2], Anja Poehlein[2], Katrin Grosser[1], Andreas Teske[3], Thorsten Brinkhoff[1]

[1] Institute for Chemistry and Biology of the Marine Environment (ICBM), University of Oldenburg, Oldenburg, Germany

[2] Department of Genomic and Applied Microbiology and Göttingen Genomics Laboratory, Institute of Microbiology and Genetics, University of Göttingen, Göttingen, Germany

[3] Department of Marine Sciences, University of North Carolina, Chapel Hill, North Carolina, United States of America

*Corresponding author: Helge-Ansgar Giebel (giebel@icbm.de)

Figure S1

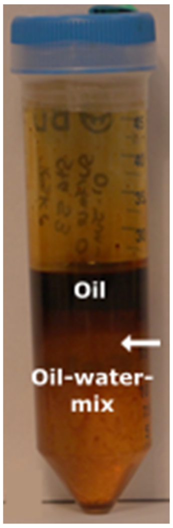

Figure S1. Enrichment culture of surface seawater contaminated with weathered oil (slicks) from the Deepwater Horizon (DWH) oil spill with oil and oil-water phase. From the latter, indicated by an arrow, *Rhodobacteraceae* strain O3.65 was isolated.

Table S1. Dissimilarity (%) based on 16S rRNA gene sequence comparison and *in silico* DNA-DNA hybridization (DDH) of strain O3.65 using 16S rRNA gene sequences and genomes of (type^{T}) strains of the genera *Phaeobacter*, *Pseudophaeobacter*, *Leisingera*, *Ruegeria* and other relevant strains. The neighbor-joining distance matrix tool of the ARB software was used for calculation of 16S rRNA gene similarity. DDH was done using the genome to genome distance calculator (GGDC 2.0, DSMZ, http://ggdc.dsmz.de/distcalc2.php, [58,59]) and represents values of the recommended formula 2 [60]. Table sorted by increasing dissimilarity.

Reference strain	GenBank accession number	Dissimilarity (%)	DDH (%)	Reference
Ruegeria sp. 39RL_GOM-46m	PRJNA213767[a]	0	100.0±0.1	-
Uncultured *alpha proteobacterium* clone Oil-BE-016	KJ475503	0.1	-	[12]
Phaeobacter inhibens DSM 17395	ABIF01000020	1.6	19.8±2.3	[71,81]
Phaeobacter gallaeciensis DSM 26640^{T}	NC_023137	1.7	21.8±2.4	[69]
Phaeobacter inhibens T5^{T}	NZ_AXBB00000000	1.7	20.2±2.3	[30]
Ruegeria scottomollicae LMG 24367^{T}	AM905330	1.9	-	[82]
Phaeobacter sp. LSS9	GQ906799	2.0	20.8±2.3	[83]
Leisingera aquimarina DSM 24565^{T}	NZ_AXBE01000000	2.0	20.1±2.3	[67]
Leisingera methylohalidivorans DSM 14336^{T}	NC_023135	2.1	20.2±2.3	[68]
Roseobacter sp. SK209-2-6	NZ_AAYC00000000	2.2	20.1±2.3	-
Phaeobacter inhibens 2.10	NC_018290	2.2	20.1±2.3	[71]
Leisingera aquaemixtae SSK6-1^{T}	KF554505	2.3	-	[32,84]
Leisingera caerulea DSM 24564^{T}	AXBI00000000	2.6	20.4±2.3	[31]
Ruegeria sp. R11	NZ_ABXM01000000	2.7	20.3±2.3	-
Ruegeria faecimaris HD-28^{T}	GU057915	2.8	-	[85]
Leisingera sp. ANG1	NZ_AFCF00000000	3.1	19.8±2.3	[86]
Leisingera daeponensis DSM 23529^{T}	NZ_AXBD00000000	3.2	20.7±2.3	
Rhodobacterales bacterium Y4I	ABXF00000000	3.2	20.4±2.3	-
Roseobacter sp. MED193	NZ_AANB01000000	3.3	21.2±2.3	-
Sedimentitalae nanhaiensis DSM 24252^{T}	NZ_AXBG00000000	3.4	22.8± 2.4	[32]
Pelagicola litoralis CL-ES2^{T}	EF192392	3.4	-	[87]
Ruegeria mobilis 45A6	PRJNA295947	3.5*	21.5±2.3	-
Ruegeria sp. TrichCH4B	NZ_ACNZ00000000	3.5	21.7±2.4	-
Pseudophaeobacter arcticus DSM 23566^{T}	NZ_AXBF00000000	3.7	20.3±2.3	[32,88]
Seohicola saemankumensis SD-15^{T}	EU221274	3.8	-	[89]
Ruegeria atlantica DSM 5823^{T}	D88526	3.9	-	[90]
Ruegeria pelagia HTCC2662^{T}	DQ916141	3.9	-	[91]
Ruegeria conchae TW15^{T}	NZ_AEYW01000000	4.2	19.2±2.3	[92]
Phaeobacter marinintestinus UB-M7	KJ461690	4.2	-	[93]
Ruegeria pomeroyi DSS-3^{T}	NC_003911.12	4.3	19.9±2.3	[94]
Rhodobacteraceae bacterium KLH11	NZ_ACCW01000000	4.3	19.3±2.3	-
Ruegeria arenilitoris G-M8^{T}	JQ807219	4.3	-	[95]
Ruegeria halocynthiae MA1-6^{T}	HQ852038	4.3	-	[96]

Ruegeria marina ZH17^T	FJ872535	4.4	20.4±2.3	[97]
Ruegeria lacuscaerulensis ITI-1157^T	NZ_ACNX01000000	4.5	20.0±2.3	-
Litorimicrobium taeanense G4^T	GQ232737	4.6	22.7±2.4	[98]
Ruegeria sp. TM1040	NC_008044	4.6	21.2±2.3	[99]
Ruegeria halocynthiae MOLA R1/13b	NZ_JQEZ00000000	4.6	19.7±2.3	[100]
Ruegeria sp. ANG-S4	NZ_JWLK01000000	4.7	19.9±2.3	[86]
Ruegeria sp. ANG-R	NZ_JWLJ01000000	4.7	18.6±2.3	[86]
Ruegeria meonggei Ma-E2-3^T	KF740534	4.9	-	[101]
Pseudophaeobacter leonis 306^T	HE661585	5.0	-	[32,102]
Ruegeria intermedia CC-GIMAT-2^T	FR832879	5.8	-	[103]

[a] bioproject number.
* 16S gene sequence identical to *Ruegeria mobilis* NBRC 101030^T (AB255401)
